# Mobility of FLOWERING LOCUS T protein as a systemic signal in trifoliate orange and its low accumulation in grafted juvenile scions

**DOI:** 10.1093/hr/uhac056

**Published:** 2022-03-07

**Authors:** Yan-Mei Wu, Yu-Jiao Ma, Min Wang, Huan Zhou, Zhi-Meng Gan, Ren-Fang Zeng, Li-Xia Ye, Jing-Jing Zhou, Jin-Zhi Zhang, Chun-Gen Hu

## Abstract

The long juvenile period of perennial woody plants is a major constraint in breeding programs. FLOWERING LOCUS T (FT) protein is an important mobile florigen signal that induces plant flowering. However, whether FT can be transported in woody plants to shorten the juvenile period is unknown, and its transport mechanism remains unclear. In this study, trifoliate orange *FT* (*ToFT*) and *Arabidopsis FT* (*AtFT*, which has been confirmed to be transportable in *Arabidopsis*) as a control were transformed into tomato and trifoliate orange, and early flowering was induced in the transgenic plants. Long-distance and two-way (upward and downward) transmission of ToFT and AtFT proteins was confirmed in both tomato and trifoliate orange using grafting and western blot analysis. However, rootstocks of transgenic trifoliate orange could not induce flowering in grafted wild-type juvenile scions because of the low accumulation of total FT protein in the grafted scions. It was further confirmed that endogenous ToFT protein was reduced in trifoliate orange, and the accumulation of the transported ToFT and AtFT proteins was lower than that in grafted juvenile tomato scions. Furthermore, the trifoliate orange *FT-INTERACTING PROTEIN1* homolog (*ToFTIP1*) was isolated by yeast two-hybrid analysis. The FTIP1 homolog may regulate FT transport by interacting with FT in tomato and trifoliate orange. Our findings suggest that FT transport may be conserved between the tomato model and woody plants. However, in woody plants, the transported FT protein did not accumulate in significant amounts in the grafted wild-type juvenile scions and induce the scions to flower.

## Teaser

FLOWERING LOCUS T (FT) protein undergoes long-distance movement as a systemic signal in transgenic and transgrafted plants, and FT-INTERACTING PROTEIN1 homolog may regulate its transport by interacting with FT.

## Introduction

The development of perennial woody plants has two distinct phases: juvenile and adult [1]. The juvenile period is the specific stage from the germination of the seed to the first flowering in perennial woody plants; it lasts for several years to decades, depending on the environment and genotype [2, 3]. During the juvenile phase, plants are unable to flower, even under inductive conditions [4, 5]. Therefore, the phase transition from juvenile to adult is actually the most important event in the entire life cycle of perennial woody plants [6, 7]. Numerous studies have found that the juvenile-to-adult transition involves developmental and environmental flowering-inducing factors [8–10]. Molecular studies have suggested that these factors can be transported over long distances in plants [11, 12]. A classic grafting experiment by Chailakhyan et al. confirmed that a graft-transmittable signaling molecule is synthesized in the stock leaf and transported to the apical meristem, where it stimulates the scion to flower [13, 14]. The molecular identity of florigen has been investigated for nearly 70 years, and florigen was eventually discovered to be the well-known FLOWERING LOCUS T (FT) protein [15]. To date, abundant experimental evidence from model plants such as *Arabidopsis*, tomato, and rice has suggested that FT, as a mobile signal, is synthesized in the leaves and transported to the apical meristem of the plant to induce flowering [12, 15–17].

In model plants, *FT* transcription has been shown to be activated in the leaf phloem by the CONSTANS (CO) protein; FT then forms a complex with a bZIP transcription factor, FD, at the shoot apical meristem (SAM) to induce the expression of the downstream floral meristem gene *LEAFY* (*LFY*) or *APETALA1* (*AP1*), thereby promoting flowering [16–18]. Although the mobility of FT is an important feature, the regulatory mechanism of FT protein transport is poorly understood. In the past decade, it has been shown that in *Arabidopsis* and rice, two proteins can interact directly with FT by regulating the long-distance transport of FT protein from leaf to SAM to regulate flowering time [19–21]. FT-INTERACTING PROTEIN 1 (FTIP1) is an endoplasmic reticulum (ER) protein that plays a key role in uploading FT from the companion cells to the sieve elements in the phloem [19]. SODIUM POTASSIUM ROOT DEFECTIVE 1 (NaKR1) encodes a protein with heavy metal-related domains that is specifically required for photoperiodic control of long-distance FT transport in the phloem stream [20]. Thus, these two proteins facilitate the regulation of FT transport. However, although the mechanism of FT transport has been well explored in model plants, the regulation of florigen transport is still unclear in other flowering plants, particularly woody perennials.

*FT* and *FT-like* genes have been isolated and functionally analyzed in numerous woody perennial plants, including poplar, apple, citrus, grape, and blueberry [22–26]. The graft-transmitted FT protein has also been extensively studied in different woody plants [24, 27–29]. However, contradictory observations on graft delivery of FT to induce flowering have been reported in some woody plants [24, 30]. Phenotypic analysis revealed graft-transmissible early flowering in *Jatropha* [30, 31] and blueberries [25], which are small woody shrubs with short juvenile stages. By contrast, grafting experiments using *FT*-transgenic poplar and apple rootstocks did not induce flowering in the receptor scions [23, 32]. In these studies, the potential transport of FT protein or RNA as a florigen signal was not investigated using western blot or northern blot analysis. Therefore, direct evidence for the possible long-distance mobility of FT proteins is very scarce in woody plants. Recently, ectopic expression of a pear FT-like protein (PcFT2) fused with yellow fluorescent protein (YFP) provided some evidence that this effect can be transported across the graft junction, and it was observed that wild-type scions grafted onto transgenic apple became insensitive to short-day-induced dormancy [29]. A recent study also demonstrated that long-range transport of *FT ortholog* (*FT2*) causes leaf-mediated seasonal control of shoot growth in poplar [27]. However, the distance traveled by FT is very short, and the signal detected is also very weak, according to previous studies on woody plants. Whether the mobile florigen signal is not the FT protein, but rather some other FT derivative with high mobility, remains to be determined in many plants, especially horticulturally important woody trees.

Citrus is one of the most popular fruit trees and an important commercial fruit crop worldwide [33]. The juvenile period of citrus ranges from 6 to 20 years, seriously hindering conventional breeding and heredity improvement. Furthermore, the selection of ideal commercial and cultural traits through traditional breeding methods is time consuming [34]. Shortening of the juvenile period and early flowering have therefore always been important breeding goals for citrus [33, 35]. Several studies have shown that citrus *FT* can shorten the juvenile period and participate in seasonal flowering [22, 36–38]. For example, three *FT* and *FT-like* genes are closely related to low temperature-induced seasonal flowering in Satsuma mandarin (*Citrus unshiu* Marc.) [38, 39]. Further functional analysis found that some of them could significantly shorten the juvenile stage of different citrus species [22, 36, 37, 40]. However, it remains unclear whether these FT proteins or mRNAs are mobile in citrus and can shorten the juvenile period.

Grafting is an important tool for improving the production of horticultural crops. For citrus cultivation, the selection of appropriate rootstocks can improve nutrient absorption, plant vitality, stress resistance, and yield [33, 41, 42]. Furthermore, precocious flowering of rootstocks carrying florigen would provide a helpful and safe tool for dramatically accelerating citrus genetic studies and breeding. Trifoliate orange (*Citrus trifoliata*) is commonly used as a rootstock in citrus cultivation, and we used it as an experimental material in this study. To understand the long-distance movement of FT protein in trifoliate orange, we generated transgenic trifoliate orange and tomato plants overexpressing *FT* from trifoliate orange (*ToFT*) and *Arabidopsis* (*AtFT*) and showed that the two FT proteins induced flowering in the two types of transgenic plants. Grafting experiments also showed that ToFT and AtFT proteins were mobile in tomato and trifoliate orange, suggesting that FT transport may be conserved among different plants. In addition, we found that ToFTIP can interact with ToFT and may participate in the long-distance transport of ToFT in trifoliate orange.

## Results

### *ToFT* and *AtFT* promote flowering in transgenic and transgrafted tomato

**Figure 1:**
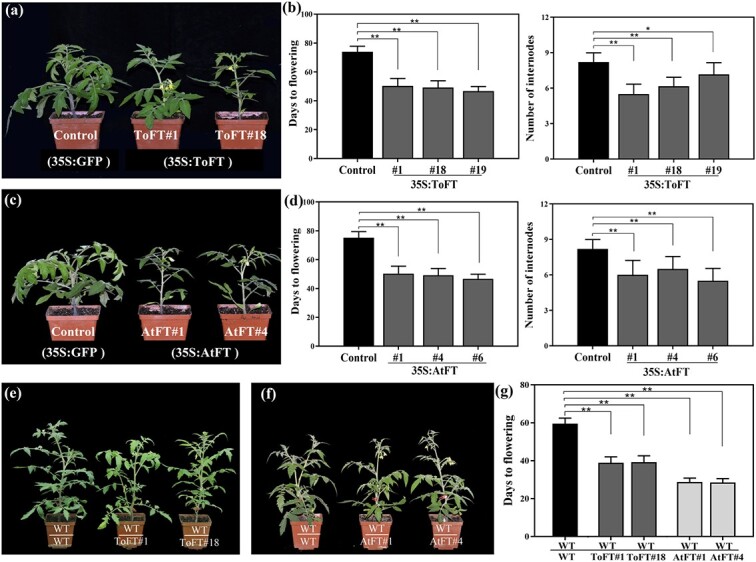
*ToFT* and *AtFT* promote early flowering in transgenic and transgrafted tomato. (a) Phenotypes of *35S:ToFT* transgenic tomato. (b) Number of days to flowering and number of internodes in *35S:ToFT* transgenic tomato and the control from seed sowing to flowering. Fifteen plants of each genotype were used for statistical analysis. Error bars represent ± SE (n = 15). (c) Phenotypes of *35S:AtFT* transgenic tomato. (d) Number of days to flowering and number of internodes in *35S:AtFT* transgenic tomato and the control from seed sowing to flowering. (e–f) Phenotype of wild-type juvenile tomato scions grafted onto *35S:ToFT* (e) and *35S:AtFT* transgenic tomato (f) rootstocks. (g) The flowering time of wild-type juvenile tomato scions grafted onto *35S:AtFT* and *35S:AtFT* transgenic tomato rootstocks. Each grafting combination included 12 individual plants for statistical analysis. Error bars represent ± SE (n = 12). ToFT#1, ToFT#18, and ToFT#19 represent three *35S:ToFT* transgenic tomato lines. AtFT#1, AtFT#4, and AtFT#6 represent three *35S:AtFT* transgenic tomato lines. Asterisks signify significant differences: ^**^P < 0.01, ^*^P < 0.05. WT/WT, WT/AtFT, and WT/ToFT represent wild-type scions grafted onto wild-type rootstock, *35S:AtFT* transgenic rootstock, and *35S:ToFT* transgenic rootstock, respectively. Note that wild-type and transgenic plants were grown under the same conditions and studied at the same developmental stage.

To investigate the biological functions of *ToFT* and *AtFT*, transgenic tomato plants were obtained by overexpression of *ToFT* and *AtFT* with a strong constitutive 35S promoter (Fig. 1a–d). The empty *GFP* vector (*35S:GFP*) was transformed into tomato as a control. More than ten transgenic lines were obtained for each gene; all the transgenic lines displayed obvious early flowering compared with the *GFP* control and the wild type. For each gene, three T_3_ homozygous transgenic lines were randomly selected for further analysis. Compared with the control, the flowering time of *ToFT* and *AtFT* transgenic plants was significantly earlier in terms of both node number and days to flowering (Fig. 1a–d). To further investigate the correlation between flowering time and the expression of *ToFT* and *AtFT* in transgenic plants, the expression levels of the tomato *FT* homolog (*SINGLE FLOWER TRUSS*: *SFT*), *LFY* homolog (*SlLFY*), and *AP1* homolog (*SlAP1*) were investigated at the flowering transition stage (Fig. S1). The results indicated that the expression levels of *SlLFY* and *SlAP1* were significantly increased in the apical meristem of transgenic plants compared with the control (Fig. S1). However, the expression of *SFT* was significantly suppressed in the leaves of *AtFT* and *ToFT* transgenic tomato (Fig. S1).

To verify whether *ToFT* is transported in tomato, wild-type tomato scions were grafted onto *35S:ToFT* and *35S:AtFT* transgenic tomato rootstocks; two T_3_ homozygous transgenic lines were selected for each type of transgenic plant (ToFT#1 and ToFT#18 for *35S:ToFT* transgenic tomato, AtFT#1 and AtFT#4 for *35S:AtFT* transgenic tomato). A total of seven grafting combinations (ToFT/WT, WT/ToFT, AtFT/WT, WT/AtFT, GFP/WT, WT/GFP, and WT/WT) were generated, each of which included 12 individual plants (Fig. 1e–g). *35S:AtFT* was used as a positive control because *AtFT* has been shown to be transportable in model plants [16], and *35S:GFP* empty vector in transgenic plants was used as a negative control. Subsequently, the flowering times of the grafted combinations were investigated. The flowering time did not differ significantly between the WT/GFP and WT/WT graft combinations. Compared with the control (WT/GFP), the transgenic rootstocks of *AtFT* and *ToFT* (WT/ToFT and WT/AtFT) significantly promoted early flowering of the wild-type scions (Fig. 1e–g). In the control grafted plants, wild-type scions were grafted onto *35S:GFP* transgenic rootstocks, and the grafted scions began to flower at 59.56 ± 2.92 days. However, when wild-type scions were grafted onto *35S:ToFT* and *35S:AtFT* transgenic rootstocks, the scions began to flower between 28.56 ± 2.01 and 39.22 ± 3.38 days (Fig. 1g). These results implied that the florigen signal originating from *ToFT* and *AtFT* may cause early flowering of wild-type scions through graft transmission. The flowering time of ToFT/WT, AtFT/WT, and GFP/WT grafting combinations was not analyzed further because the new shoot budding time of the wild-type rootstocks is different. These graft combinations were used for the FT protein transport analysis.

### ToFT and AtFT proteins are transported from transgenic rootstocks to wild-type scions in tomato

**Figure 2:**
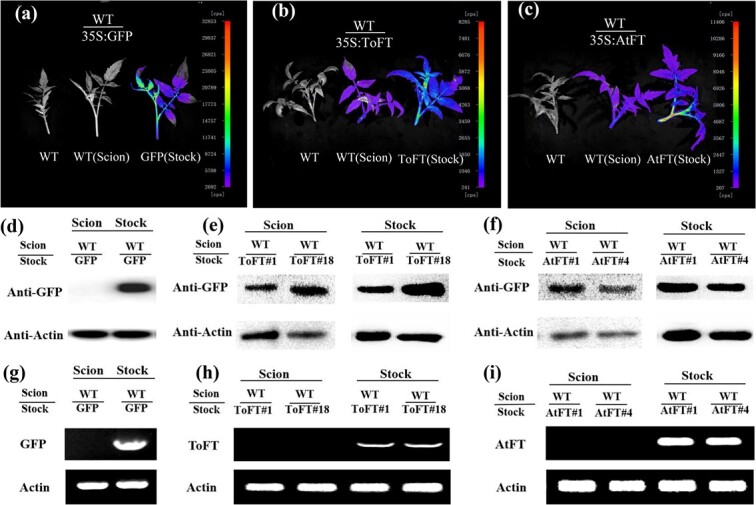
ToFT and AtFT proteins are transported from transgenic rootstocks to wild-type scions in tomato. (a–c) Detection of the movement of GFP (a), ToFT-GFP (b), and AtFT-GFP (c) proteins with the NightSHADE LB 985 imaging system; *35S:GFP*, *35S:ToFT-GFP*, and *35S:AtFT-GFP* transgenic tomato were used as rootstocks. (d–f) Detection of the movement of GFP (d), ToFT-GFP (e), and AtFT-GFP (f) proteins by western blot analysis; *35S:GFP*, *35S:ToFT-GFP*, and *35S:AtFT-GFP* transgenic tomato were used as rootstocks. (g–i) Detection of the movement of *GFP* (g), *ToFT-GFP* (h), and *AtFT-GFP* (i) mRNAs by RT–PCR analysis; *35S:GFP*, *35S:ToFT-GFP*, and *35S:AtFT-GFP* transgenic tomato were used as rootstocks. WT/GFP, WT/AtFT, and WT/ToFT represent wild-type scions grafted onto *35S:GFP*, *35S:AtFT*, and *35S:ToFT* transgenic rootstocks, respectively. GFP (Stock), AtFT (Stock), and ToFT (Stock) represent *35S:GFP*, *35S:AtFT*, and *35S:ToFT* transgenic tomato as rootstocks, respectively. AtFT#1 and AtFT#4 represent two *35S:AtFT* transgenic tomato lines. ToFT#1 and ToFT#18 represent two transgenic tomato lines. Note that wild-type and transgenic plants were grown under the same conditions and studied at the same developmental stage.

In model plants, FT protein is a mobile flowering signal [16]. *35S:AtFT* and *35S:ToFT* transgenic tomato rootstocks promoted flowering in wild-type juvenile tomato scions (Fig. 1e–g). We speculated that ToFT and AtFT proteins or mRNAs may promote early flowering of wild-type juvenile scions through graft transmission. In this study, ToFT and AtFT were fused with the GFP protein and then transformed into tomato. Therefore, we first investigated whether ToFT and/or AtFT proteins were transferred using the NightSHADE LB 985 imaging system. Fig S2a shows the grafting method and sampling location. The length of the wild-type scion was 20–30 cm at the observation stage (3 weeks after grafting). When the wild-type scions were grafted onto *35S:ToFT* and *35S:AtFT* transgenic rootstocks, transport of ToFT-GFP and AtFT-GFP proteins through the tomato graft unions to wild-type scions was observed at the top of the wild-type tomato scions (Fig. 2a–c). However, no GFP protein transport was observed in wild-type tomato scions that were grafted onto *35S:GFP* control transgenic rootstocks (Fig. 2a).

To further investigate whether the ToFT-GFP and AtFT-GFP proteins were transported, western blotting was performed with a GFP antibody (Fig. 2d–f), and apical meristems including leaves of the wild-type scions above the graft union were used for total protein extraction (Fig. S2a). The results of western blot analysis showed that ToFT-GFP and AtFT-GFP proteins were present in the grafted scions (Fig. 2d–f). By contrast, we did not detect GFP protein in the control scions when wild-type scions were grafted onto *35S:GFP* transgenic rootstocks (Fig. 2d). In *Arabidopsis*, long-distance transport of *AtFT* RNA also plays a part in systemic floral regulation [43]. Therefore, we further investigated whether *ToFT* and/or *AtFT* mRNAs were transferred using RT-PCR (Fig. 2g–i). Apical meristems including leaves of the wild-type scions were sampled as described previously for western blot analysis (Figure S2a). However, no *ToFT* or *AtFT* mRNA was detected in these grafted scions (Fig. 2g–i), and *GFP* mRNA was not detected when the wild-type scion was grafted onto the *35S:GFP* transgenic rootstock (Fig. 2g). These results indicate that the mobile florigen signal from ToFT and AtFT in transgenic tomato is a protein rather than an mRNA, which is consistent with the results of the SFT protein in tomato [12].

### ToFT and AtFT proteins are transported from transgenic scions to wild-type rootstocks in tomato

**Figure 3:**
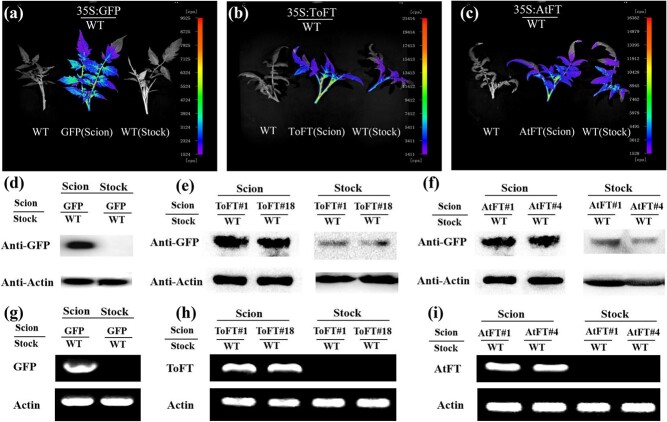
ToFT and AtFT proteins are transported from transgenic scions to wild-type rootstocks in tomato. (a–c) Detection of the movement of GFP (a), ToFT-GFP (b), and AtFT-GFP (c) proteins with the NightSHADE LB 985 imaging system. (d–f) Detection of the movement of GFP (d), ToFT-GFP (e), and AtFT-GFP (f) proteins by western blot analysis. (g–i) Detection of the movement of *GFP* (g), *ToFT-GFP* (h), and *AtFT-GFP* (i) mRNAs by RT–PCR analysis. GFP/WT, AtFT/WT, and ToFT/WT represent *35S:GFP*, *35S:AtFT*, and *35S:ToFT* transgenic scions grafted onto wild-type rootstocks, respectively. GFP (Scion), AtFT (Scion), and ToFT (Scion) represent *35S:GFP*, *35S:AtFT*, and *35S:ToFT* scions of transgenic plants, respectively. AtFT#1 and AtFT#4 represent two *35S:AtFT* transgenic tomato lines. ToFT#1 and ToFT#18 represent two transgenic tomato lines. Note that wild-type and transgenic plants were grown under the same conditions and studied at the same developmental stage.

To further investigate whether the transport of FT protein is bi-directional, the transport of ToFT and AtFT proteins from the transgenic scions to the wild-type rootstocks was also investigated. Fig. S2b shows the grafting method and the location of the collected sample. The results obtained from the NightSHADE LB 985 imaging system showed the presence of ToFT-GFP and AtFT-GFP proteins in shoots originating from wild-type rootstocks (Fig. 3a–c). As expected, ToFT-GFP and AtFT-GFP proteins were also detected by western blot analysis (Fig. 3d–f). Similar to previous results, *ToFT-GFP* and *AtFT-GFP* mRNAs could not be detected by RT-PCR analysis (Fig. 3g–i). When *35S:GFP* transgenic tomato was used as the scion and grafted onto the wild-type rootstock, no transported GFP protein or mRNA was detected (Fig. 3d, g). Regarding FT movement in tomato, the GFP signal could be detected in the apical meristem 30 cm above the graft union after 3 weeks when wild-type scions were grafted onto transgenic rootstocks. Interestingly, the GFP signal was also observed in the roots of wild-type rootstocks when *35S:ToFT* or *35S:AtFT* transgenic scions were grafted onto the wild-type rootstocks (Fig. S3). These results demonstrated that the functions of ToFT and AtFT may be mediated by systemic signals.

### *ToFT* and *AtFT* promote early flowering in transgenic trifoliate orange

**Figure 4:**
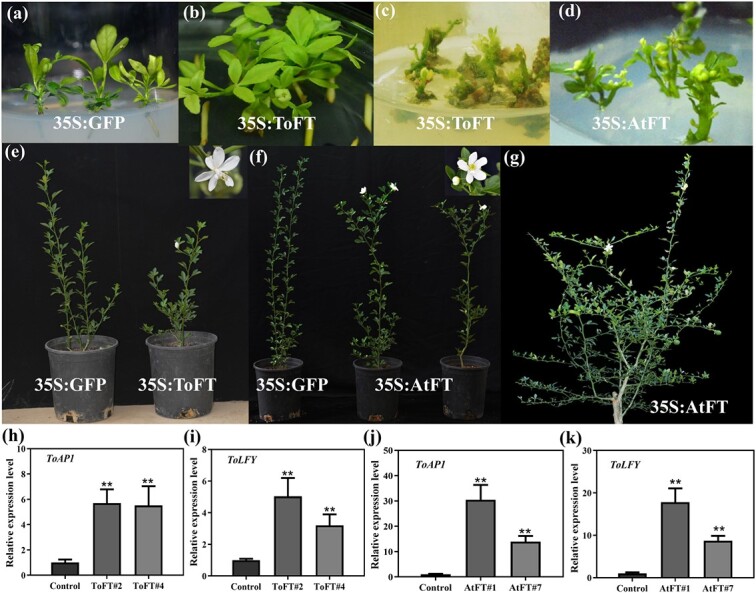
Phenotype analysis of *ToFT* and *AtFT* transgenic trifoliate orange. (a) *35S:GFP* transgenic trifoliate orange. (b–c) Extremely early flowering in *35S:ToFT* transgenic trifoliate orange. (d) Extremely early flowering in *35S:AtFT* transgenic trifoliate orange. (e) Early flowering in *35S:ToFT* transgenic trifoliate orange after transfer to the greenhouse. (f) Early flowering in *35S:AtFT* transgenic trifoliate orange after transfer to the greenhouse. (g) *35S:AtFT* transgenic trifoliate orange flowered several times a year. (h–i) Expression analysis of *ToLFY* (h) and *ToAP1* (i) in *35S:ToFT* transgenic trifoliate orange. (j–k) Expression analysis of *ToLFY* (j) and *ToAP1* (k) in *35S:AtFT* transgenic trifoliate orange. Error bars represent ± SE (n = 3). Asterisks indicate significant differences: ^**^P < 0.01. AtFT#1 and AtFT#7 represent two *35S:AtFT* transgenic trifoliate orange lines. ToFT#2 and ToFT#4 represent two *35S:ToFT* transgenic trifoliate orange lines.

To further determine whether ToFT and AtFT proteins are mobile in trifoliate orange, we introduced *ToFT-GFP* and *AtFT-GFP* into trifoliate orange with the 35S promoter (Fig. 4). The *GFP* empty vector (*35S:GFP*) was also transformed into trifoliate orange as a control. Six and nine independent *35S:ToFT-GFP* and *35S:AtFT-GFP* transgenic lines were generated, respectively. For *35S:ToFT* and *35S:AtFT* transgenic trifoliate orange, there was a large difference in flowering time among the different transgenic lines. Some transgenic lines (three *35S:ToFT* transgenic lines and four *35S:AiFT* transgenic lines) began to form flowers at the tissue culture stage (Fig. 4b–c). However, these extremely early flowering transgenic lines with abnormal flowers did not form roots and complete plants (Fig. S4). In contrast to the control plants, the other transgenic lines flowered less than 10 months after being transferred to the greenhouse (Fig. 4e–g). There was a large difference in the position of flower bud formation between transgenic and wild-type trifoliate orange (Fig. 4e–g). In transgenic plants, the apical meristem of most vegetative shoots eventually terminated with a flower, and other buds rarely formed flower buds (Fig. 4e–f). However, the terminal bud and the following five buds from spring shoots are the major node locations for flower formation in wild-type trifoliate orange [44]. Interestingly, *35S:AtFT* transgenic plants flowered several times a year (Fig. 4g), similar to previous studies on apple, pear, and olive [45–48].

To further investigate the correlation between flowering time and the expression of *35S:ToFT* and *35S:AtFT* in transgenic trifoliate orange, the expression levels of *ToLFY* and *ToAP1* were investigated in these plants (Fig. 4h–k). Levels of *ToLFY* and *ToAP1* expression were clearly increased in the new shoot apical meristems of *35S:AtFT* and *35S:ToFT* transgenic plants compared with the controls (Fig. 4h–k). Our results indicate that *ToFT* and *AtFT* can markedly accelerate flowering in trifoliate orange.

### ToFT and AtFT proteins are transported from transgenic rootstocks to wild-type scions in trifoliate orange

To investigate whether the movement of ToFT and AtFT proteins or mRNAs also occurs in trifoliate orange, we grafted wild-type trifoliate orange scions onto *35S:ToFT* and *35S:AtFT* transgenic rootstocks in autumn by bud grafting. Two transgenic lines (ToFT#2 and ToFT#4 for *ToFT* transgenic trifoliate orange and AtFT#1 and AtFT#7 for *35S:AtFT* transgenic trifoliate orange) were selected for each transgenic gene, and each grafting combination included 12 grafted plants. A total of seven graft combinations were generated (ToFT/WT, WT/ToFT, AtFT/WT, WT/AtFT, GFP/WT, WT/GFP, and WT/WT). In general, floral induction in adult trifoliate orange is initiated in the late spring and early summer in Wuhan, China, and flower buds develop after floral induction [44, 49]. Therefore, spring shoots from the grafted plants were used for the movement analysis. For floral bud initiation of trifoliate orange, self-pruning of spring shoots is necessary but not sufficient [44]. In this study, the leaves of spring shoots from different graft combinations were sampled after self-pruning for total protein and RNA extraction.

**Figure 5:**
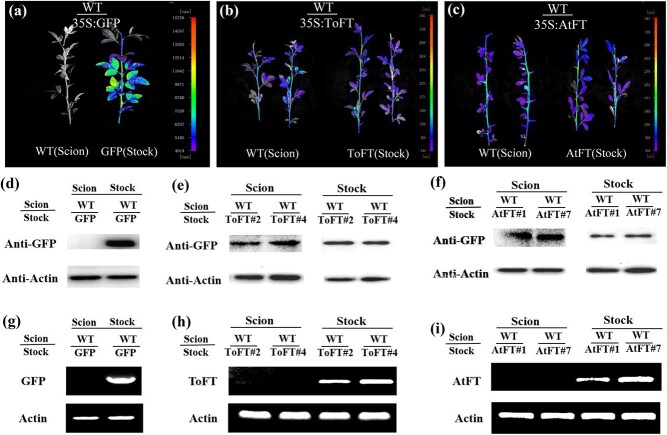
ToFT and AtFT proteins are transported from transgenic rootstocks to wild-type scions in trifoliate orange. (a–c) Detection of the movement of GFP (a), ToFT-GFP (b), and AtFT-GFP (c) proteins with the NightSHADE LB 985 imaging system. (d–f) Detection of the movement of GFP (d), ToFT-GFP (e), and AtFT-GFP (f) proteins by western blot analysis. (g–i) Detection of the movement of *GFP* (g), *ToFT-GFP* (h), and *AtFT-GFP* (i) mRNAs by RT–PCR analysis. WT/GFP, WT/AtFT, and WT/ToFT represent wild-type scions grafted onto *35S:GFP*, *35S:AtFT*, and *35S:ToFT* transgenic rootstocks, respectively. GFP (Stock), AtFT (Stock), and ToFT (Stock) represent *35S:GFP*, *35S:AtFT*, and *35S:ToFT* transgenic trifoliate orange as rootstocks, respectively. AtFT#1 and AtFT#7 represent two *35S:AtFT* transgenic trifoliate orange lines. ToFT#2 and ToFT#4 represent two transgenic trifoliate orange lines. Note that wild-type and transgenic plants were grown under the same conditions and studied at the same developmental stage.

The transport of ToFT-GFP and AtFT-GFP proteins was first investigated in these grafting combinations using the NightSHADE LB 985 imaging system. Fig S5a shows the grafting method and sampling location. Similar to tomato grafting, grafting experiments in trifoliate orange showed that ToFT-GFP and AtFT-GFP signals could be clearly detected in wild-type scions grafted onto *35S:ToFT* or *35S:AtFT* transgenic trifoliate orange rootstocks (Fig. 5a–c). For FT movement in trifoliate orange, the GFP signal could still be detected at the top, 1.5 m above the graft union (Fig. S6). The movement of ToFT-GFP and AtFT-GFP fusion proteins through graft unions from transgenic trifoliate orange rootstocks to wild-type scions was also detected by western blotting (Fig. 5d–f). These results confirm that the FT protein can be transported upward in trifoliate orange. In addition, we examined the movement of *ToFT-GFP* and *AtFT-GFP* mRNAs through graft unions using RT-PCR (Fig. 5g–i). To distinguish mRNA of the transgene from that of endogenous *ToFT*, PCR was performed with gene-specific primers for the *FT-GFP* fusion gene in *ToFT*-transgenic trifoliate orange. However, transfer of *ToFT* or *AtFT* mRNAs from transgenic rootstocks to wild-type scions was not detected (Fig. 5g–i). Likewise, when *35S:GFP* transgenic trifoliate orange was used as the scion and grafted onto wild-type rootstocks, no transported GFP protein or mRNA was detected (Fig. 5d, g). These results indicate that ToFT and AtFT proteins may be systematic signals and can be transported over long distances in grafted trifoliate orange.

### ToFT and AtFT proteins are transported from transgenic scions to wild-type rootstocks in trifoliate orange

**Figure 6:**
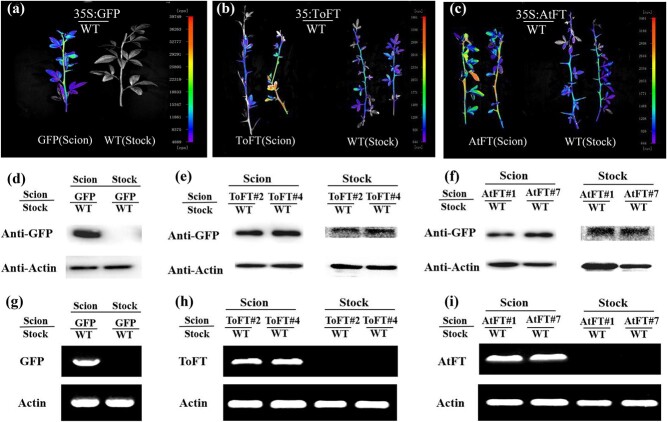
ToFT and AtFT proteins are transported from transgenic scions to wild-type rootstocks in trifoliate orange. (b–d) Detection of the movement of GFP (b), ToFT-GFP (c), and AtFT-GFP (d) proteins by the NightSHADE LB 985 imaging system. (e–g) Detection of the movement of GFP (e), ToFT-GFP (f), and AtFT-GFP (g) proteins by western blot analysis. (h–j) Detection of the movement of *GFP* (h), *ToFT-GFP* (i), and *AtFT-GFP* (j) mRNAs by RT–PCR analysis. PCR was performed with gene-specific primers for the *FT-GFP* fusion gene to distinguish it from endogenous *FT* mRNA in *35S:ToFT* transgenic trifoliate orange. GFP/WT, AtFT/WT, and ToFT/WT represent *35S:GFP*, *35S:AtFT*, and *35S:ToFT* transgenic scions grafted onto wild-type rootstocks, respectively. GFP (Scion), AtFT (Scion), and ToFT (Scion) represent *35S:GFP*, *35S:AtFT*, and *35S:ToFT* transgenic plants as scions, respectively. AtFT#1 and AtFT#7 represent two *35S:AtFT* transgenic trifoliate orange lines. ToFT#2 and ToFT#4 represent two transgenic trifoliate orange lines. Note that wild-type and transgenic plants were grown under the same conditions and studied at the same developmental stage.

To further investigate whether FT protein transport is bi-directional in trifoliate orange, similar to that in tomato, we also investigated the transport of FT protein from the transgenic scions to the wild-type rootstocks. Fig S5b shows the grafting method and sampling location. When *35S:ToFT* or *35S:AtFT* transgenic scions were grafted onto wild-type rootstocks, GFP signals were detected in the wild-type rootstocks by the NightSHADE LB 985 imaging system, suggesting movement of the ToFT-GFP or AtFT–GFP proteins from transgenic scions to wild-type rootstocks (Fig. 6a–c). The GFP signal could also be detected in the roots of wild-type rootstocks when transgenic scions were grafted onto wild-type rootstocks (Fig. S6). The movement of ToFT-GFP and AtFT-GFP proteins through graft unions to wild-type trifoliate orange rootstocks was also detected by western blotting (Fig. 6d–f). In the GFP/WT control, no GFP protein or mRNA transport was observed in the rootstocks (Fig. 6d, g). These results further confirm that the FT protein can be transported downwards in trifoliate orange.

### Transferred FT cannot induce flowering in the juvenile trifoliate orange scion

**Figure 7:**
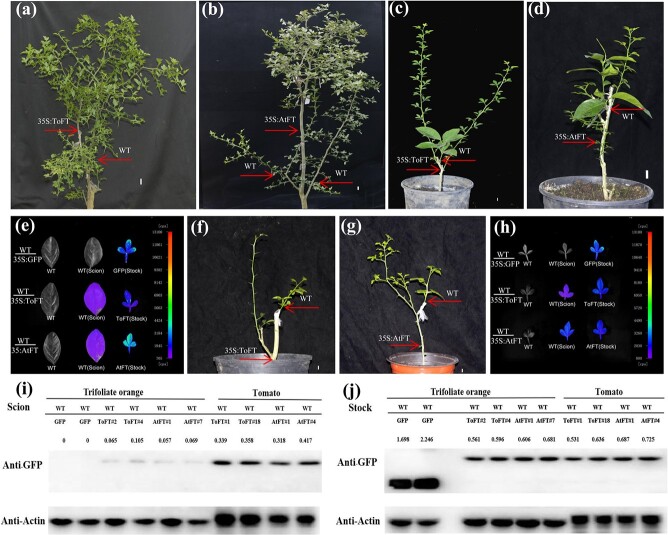
Transferred FT protein cannot induce flowering in the juvenile trifoliate orange scion. (a–b) Phenotypes of wild-type juvenile trifoliate orange scions grafted onto *35S:ToFT* (a) and *35S:AtFT* (b) transgenic rootstocks 2 years after grafting. (c–d) Phenotypes of wild-type juvenile sweet orange scions grafted onto *35S:ToFT* (c) and *35S:AtFT* (d) transgenic rootstocks 6 months after grafting. (e) The NightSHADE LB 985 imaging system detected the transfer of ToFT and AtFT proteins from transgenic rootstocks to leaves of wild-type sweet orange scions. (f–g) Phenotypes of wild-type adult trifoliate orange scions grafted onto *35S:ToFT* (f) and *35S:AtFT* (g) transgenic rootstocks 2 months after grafting. (h) The NightSHADE LB 985 imaging system detected the transfer of ToFT and AtFT proteins from transgenic rootstocks to leaves of wild-type scions. (i) Comparative analysis of the accumulation of ToFT and AtFT proteins in grafted wild-type trifoliate orange and tomato scions by western blotting. (j) Comparative analysis of the expression of GFP, ToFT, and AtFT proteins in the *35S:GFP*, *35S:AtFT*, and *35S:ToFT* transgenic rootstocks by western blotting. AtFT#1 and AtFT#7 represent two *35S:AtFT* transgenic trifoliate orange lines. ToFT#2 and ToFT#4 represent two *35S:ToFT* transgenic trifoliate orange lines. WT/GFP, WT/AtFT, and WT/ToFT represent wild-type scions grafted onto *35S:GFP*, *35S:AtFT*, and *35S:ToFT* transgenic rootstocks, respectively. Bar = 1 cm.

To investigate whether *FT* transgenic rootstocks can promote flowering in non-transgenic trifoliate orange scions by bud grafting, a total of 84 grafted plants were investigated from seven grafting combinations, including *35S:GFP* control plants. All grafted plants were grown in a greenhouse and were healthy. However, no early flowering phenotype was observed in any wild-type juvenile scions grafted onto *35S:ToFT* and *35S:AtFT* trifoliate orange transgenic rootstocks (WT/ToFT and WT/AtFT) during the 2-year observation period (Fig. 7a–b). Juvenile Valencia orange scions were grafted onto the transgenic rootstocks of *35S:AtFT* and *35S:ToFT* (Fig. 7c–d), and movement of ToFT-GFP and AtFT-GFP proteins through the graft unions to non-transgenic scions was again detected in leaves of the wild-type scions (Fig. 7e). However, the results were similar to those of the juvenile scions of wild-type trifoliate orange grafted onto *35S:AtFT* and *35S:ToFT* transgenic rootstocks (Fig. 7a–b), and the non-flowering phenotype was observed in the grafted sweet orange scions (Fig. 7c–d). These results further indicate that *35S:AtFT* and *35S:ToFT* transgenic rootstocks may not significantly shorten the juvenile period of juvenile scions through grafting. To further investigate whether the transport of FT protein can promote the flowering of adult trifoliate orange and overcome the limitation of seasonal flowering, adult trifoliate orange was grafted onto *FT*-transgenic rootstock by the branch grafting method (Fig. 7f–g). The transport of ToFT-GFP and AtFT-GFP proteins was also observed in these grafting combinations using the NightSHADE LB 985 imaging system (Fig. 7h), but early flowering was not observed in these grafting combinations (Fig. 7f–g).

The transgenic rootstocks of *35S:AtFT* and *35S:ToFT* induced flowering in the wild-type scions in tomato (Fig. 1k–n). In addition, the movement of AtFT and ToFT proteins was also detected in grafted plants (Figs. 2–3). We speculated that the transported AtFT and ToFT proteins accumulated to greater amounts in tomato than in trifoliate orange and thus induced flowering in the grafted tomato scions. Furthermore, when investigating whether AtFT and ToFT proteins could be transported in trifoliate orange and tomato by grafting, the transported FT protein required further enrichment in trifoliate orange using an Anti-GFP Magarose Beads Kit (Fig. 7k–n). However, the transported FT protein did not require such enrichment in tomato (Figs. 2–3). To confirm our hypothesis, we further investigated the accumulation of transported AtFT and ToFT proteins in the two species by western blotting (Fig. 7i) and found that the accumulation of the transported AtFT and ToFT proteins was indeed higher in the leaves of tomato than in those of trifoliate orange at the same position (30 cm above the graft union) (Fig. 7i). To exclude the possibility that the low accumulation of transported FT protein in the scion was caused by differential expression of exogenous FT in the transgenic rootstocks, the exogenous FT protein was also investigated in different transgenic rootstocks by western blotting. The results showed that the exogenous FT did not show significant differences in the different transgenic rootstocks (Fig. 7j). These results suggested that the low accumulation of transported FT protein may be caused by different transport efficiencies of FT in different species.

### Transport of FT may inhibit the endogenous FT protein

**Figure 8:**
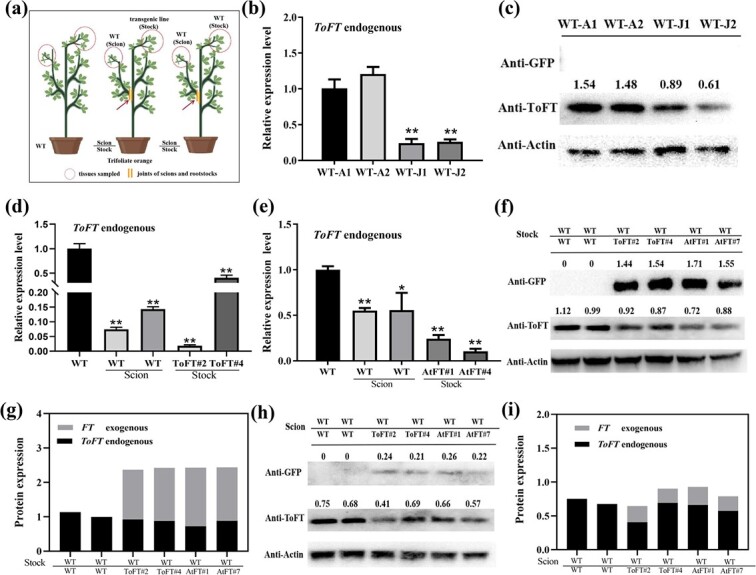
Transport of ToFT and AtFT may inhibit the endogenous FT protein. (a) The location of the collected sample in trifoliate orange. (b) Expression analysis of *ToFT* in adult and juvenile trifoliate orange by real-time PCR. WT-J1 and WT-J2 represent two groups of different juvenile trifoliate orange. WT-A1 and WT-A2 represent two groups of different adult trifoliate orange. (c) Expression analysis of ToFT in adult and juvenile trifoliate orange by western blotting using ToFT antibody. (d) The expression of endogenous *ToFT* in wild-type scions and *35S:ToFT* transgenic rootstocks when wild-type scions were grafted onto *35S:ToFT* transgenic rootstocks. (e) The expression of endogenous *ToFT* in wild-type scions and *35S:AtFT* transgenic rootstocks when wild-type scions were grafted onto *35S:AtFT* transgenic rootstocks. (f) Expression analysis of endogenous and exogenous ToFT proteins in wild-type and transgenic rootstocks when wild-type scions were grafted onto transgenic rootstocks. (g) Relative endogenous ToFT and exogenous FT protein levels based on western blotting results. (h) Expression analysis of endogenous ToFT and exogenous FT proteins in scions when wild-type scions were grafted onto transgenic rootstocks. (i) Relative endogenous ToFT and exogenous FT protein levels in scions based on western blot results. AtFT#1 and AtFT#7 represent two *35S:AtFT* transgenic trifoliate orange lines. ToFT#2 and ToFT#4 represent two *35S:ToFT* transgenic trifoliate orange lines. WT/AtFT, WT/ToFT, and WT/WT represent wild-type scions grafted onto *35S:AtFT* transgenic, *35S:ToFT* transgenic, and wild-type rootstocks. Error bars represent ± SE (n = 3). Asterisks indicate significant differences: ^**^P < 0.01, ^*^P < 0.05. Note that wild-type and transgenic plants were grown under the same conditions and studied at the same developmental stage.

To further investigate why transported ToFT or AtFT protein failed to induce flowering in juvenile trifoliate orange scions, the expression of *ToFT* in the leaves of spring shoots was investigated in adult and juvenile trifoliate orange by real-time PCR and western blotting analysis (Fig. 8a). The leaves of spring shoots were sampled after self-pruning, as described previously for the movement analysis assay (Fig. 8a). The results showed that the expression of *ToFT* in trifoliate orange was much higher in the adult stage than in the juvenile stage (Fig. 8b–c). Furthermore, overexpression of *ToFT* can significantly shorten the juvenile stage of trifoliate orange. These results suggest that the expression level of *ToFT* may be closely related to the phase transition of trifoliate orange, which is consistent with previous studies on citrus [22, 37]. The expression of endogenous *ToFT* was also investigated in these grafted plants (Fig. 8d–e). In different graft combinations of *35S:AtFT* and *35S:ToFT* transgenic trifoliate orange, the expression of endogenous *ToFT* was reduced in the recipients and donors compared with the controls (Fig. 8d–e). To distinguish exogenous *ToFT* in the graft combination from endogenous *ToFT* in transgenic trifoliate orange, one of the PCR primers was located at the 3′ UTR of *ToFT* because the exogenous *ToFT* does not contain the 5′ UTR in the *ToFT-GFP* fusion gene. Western blotting analysis with ToFT antibody showed that the endogenous ToFT protein was decreased in both *35S:AtFT* and *35S:ToFT* transgenic plants (Fig. 8f–g). Also, the total FT protein was significantly higher in transgenic plants than in the control plants (Fig. 8f–g). Therefore, we speculated that the transported ToFT or AtFT protein may inhibit the expression of endogenous ToFT.

To further verify this hypothesis, the expression of ToFT protein in the leaves of juvenile trifoliate orange (the control) and juvenile scions grafted onto *35S:ToFT* or *35S:AtFT* transgenic trifoliate orange was investigated by western blotting using ToFT and GFP antibodies (Fig. 8h–i). Compared with juvenile trifoliate orange, grafted scions showed significantly reduced endogenous ToFT protein when wild-type juvenile scions were grafted onto *35S:AtFT* or *35S:ToFT* transgenic rootstocks (Fig. 8h–i). The total ToFT protein did not accumulate in significant amounts in the grafted scions (Fig. 8h–i), although the ToFT or AtFT protein from the rootstocks was transported to the juvenile scions. These results implied that the exogenous AtFT or ToFT protein may cause a decrease in the endogenous ToFT protein, resulting in the failure of the total ToFT protein to reach the threshold required for trifoliate orange flowering.

### FTIP1 interacts with FT in tomato and trifoliate orange

**Figure 9:**
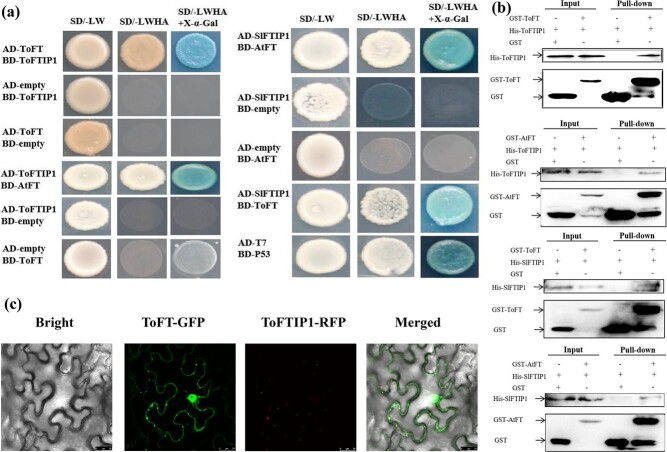
FTIP1 interacts with FT in tomato and trifoliate orange. (a) Yeast two-hybrid assay of the interactions of ToFTIP1 with AtFT, ToFTIP1 with ToFT, SlFTIP1 with AtFT, and SlFTIP1 with ToFT. Transformed yeast cells harboring FTIP1 protein and FT were incubated on SD/−Leu/−Trp (SD/-LW), SD/−Leu/−Trp/−His/−Ade (SD/-LWHA), and SD/−Leu/−Trp/−His/−Ade medium supplemented with X-α-Gal. Empty refers to the pGADT7 or pGBKT7 empty vector only. (b) GST pull-down assay confirming the interactions of ToFTIP1 with AtFT, ToFTIP1 with ToFT, SlFTIP1 with AtFT, and SlFTIP1 with ToFT. (d) Colocalization of GFP-ToFTIP1 and RFP-ToFT in tobacco leaf cells by the IntF2A-based polyprotein transgene system. Yellow color indicates the YFP fluorescence. Red color indicates the RFP fluorescence. Merge indicates the merged GFP and RFP images. Scale bar = 25 μm.

To gain insight into the mechanism by which ToFT transport is regulated, we screened a trifoliate orange yeast two-hybrid (Y2H) library from the stems of trifoliate orange using ToFT protein as bait, and a protein (LOC18044269) containing multiple C2 and transmembrane domains was identified (Fig. 9a). Sequence analysis revealed that LOC18044269 encodes 815 amino acids with three C2 domains and one PRT_C domain. Multiple sequence alignment and phylogenetic tree analysis revealed that the encoded protein of LOC18044269 shared 71% and 73% identity with *Arabidopsis* FTIP1 and rice FTIP1, respectively. These data suggested that LOC18044269 may be the putative ortholog of *FTIP1* in trifoliate orange, and it was named *ToFTIP1*. To assess the subcellular localization of ToFTIP1, we transiently expressed 35S:ToFTIP1-GFP in tobacco leaves and found that ToFTIP1-GFP was mainly localized with an ER marker (Fig. S7), consistent with research in *Arabidopsis* and rice [19, 21].

To investigate whether ToFTIP1 and ToFT interact with each other in trifoliate orange, we co-expressed ToFT-RFP and ToFTIP1-GFP in tobacco leaf cells using the IntF2A-based polyprotein transgene system [50] and found that ToFT-RFP and ToFTIP1-GFP were colocalized in the ER, which is connected to the nuclear envelope (Fig. 9c). Pull-down assays demonstrated that ToFTIP1 interacted with ToFT (Fig. 9b). A BiFC assay also revealed localization of the GFP fluorescence signal with ER-ToFTIP1 in tobacco leaf cells, indicating an interaction between ToFTIP1 and ToFT (Fig. S8). Grafting analysis has shown that AtFT can also be transported in trifoliate orange; therefore, to investigate whether the transport of AtFT protein also requires ToFTP1 in trifoliate orange, the interaction between ToFTP1 and AtFT proteins was analyzed. The protein interaction between ToFTIP1 and AtFT was confirmed by Y2H, BiFC, and pull-down assays (Fig. 9a–b and S9).

Grafting experiments showed that ToFT and AtFT proteins were transported in tomato and promoted scion flowering, suggesting that the tomato FTIP1 homolog (SlFTIP1) may play a key role in FT transport in tomato. Therefore, the interaction of SlFTIP1 with AtFT and ToFT was also investigated. We searched the tomato genome database using trifoliate orange and *Arabidopsis FTIP1* and found a tomato *FTIP1* homolog (LOC101245336). Subsequently, we used Y2H assays to examine whether AtFT and ToFT could interact with tomato SlFTIP1 in vitro. The results showed that AtFT and ToFT interacted with SlFTIP1 in yeast (Fig. 9a). These interactions were further confirmed using BiFC and pull-down assays (Fig. 9b–c). These data indicate that FTIP1 interacts with FT and may mediate its transport in tomato and trifoliate orange.

### Expression and functional analysis of *ToFTIP1*

**Figure 10:**
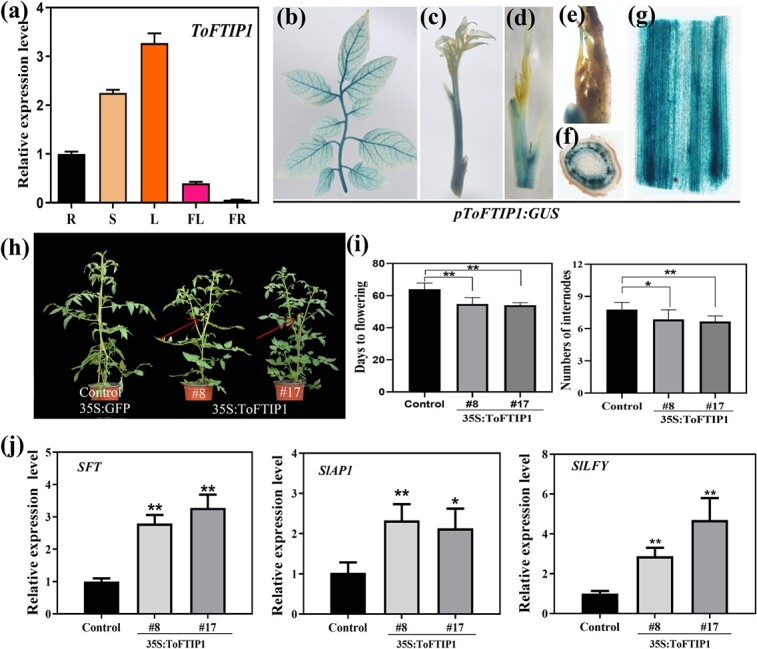
Expression and functional analysis of *ToFTIP1*. (a) Real-time PCR analysis of *ToFTIP* expression in various tissues of adult trifoliate orange. Roots (R), stems (S), leaves (L), flowers at anthesis (FL), and whole fruits at 30 days after flowering (FR). Error bars represent ± SE (n = 3). (b–g) Histochemical GUS staining of the *ToFTIP1* promoter (*pToFTIP1:GUS*) in transgenic tomato, including leaves (b), stems (c), top bud (d), SAM (e), cross section (f), and longitudinal section (g) of the stem. (h) Phenotypes of *35S:ToFTIP1* transgenic tomato and *GFP* control plants. (i) Number of days to flowering and number of internodes in *35S:ToFTIP1* transgenic tomato and the *35S:GFP* control at the flowering stage. (j) Expression analysis of tomato flowering-related genes, including *SFT, SlLFY,* and *SlAP1* in *35S:ToFTIP1* transgenic tomato. Asterisks indicate significant differences: ^**^P < 0.01, ^*^P < 0.05. ToFTIP1#8 and ToFTIP1#17 represent two *35S:ToFTIP1* transgenic tomato lines.

To characterize the functions of *ToFTIP1*, its expression pattern was investigated in various tissues of trifoliate orange using qRT-PCR (Fig. 10a). *ToFTIP1* was ubiquitously expressed in all tissues, with the highest expression in leaves and stems, similar to the pattern of *ToFT* expression [51, 52]. To further investigate the detailed expression pattern of *ToFTIP1*, we fused its promoter with a *GUS* reporter gene (*pToFTIP1:GUS*), and the construct was transformed into tomato. A total of 12 transgenic tomato lines were obtained, most of which showed similar *GUS* staining patterns (Fig. 10b–g). *PToFTIP1:GUS* was specifically expressed in the vascular tissues of various plant organs, including leaves (Fig. 10b–g), similar to the expression pattern of *FTIP1* reported in *Arabidopsis* and rice [19, 21], suggesting that *ToFTIP1* may regulate the transport of ToFT protein during trifoliate orange flowering.

To further investigate the function of *ToFTIP1*, it was transformed into tomato, and a total of 16 *35S:ToFTIP1* transgenic lines were obtained. Two T_3_ homozygous transgenic lines were randomly selected for further analysis. These transgenic lines flowered significantly earlier than the control based on the number of internodes and days to flowering (Fig. 10h). The control and transgenic plants flowered at 63.5 ± 3.84 days with 7.78 ± 0.67 internodes and 54.86 ± 3.89 days with 6.85 ± 0.90 internodes, respectively (Fig. 10i). To further investigate the correlation between flowering time and the expression of *ToFTIP1* in transgenic plants, the expression levels of tomato *SFT*, *SlLFY*, and *SlAP1* were also investigated at the flowering transition stage (Fig. 10j). The expression of *SFT* was significantly upregulated in the leaves of transgenic plants compared with those of controls (Fig. 10j). Furthermore, the expression of *SlLFY* and *SlAP1* was significantly upregulated in the apical meristems of transgenic plants (Fig. 10j). These results further suggest that *ToFTIP1* may promote trifoliate orange flowering.

## Discussion

*FT* is an evolutionarily conserved gene that is ubiquitous in almost all flowering plants [53]. Previous studies in annual plants have confirmed that FT protein acts as a florigen, a mobile flower-inducing signal that is synthesized in leaves and then transported to meristems [17, 53, 54]. Homologs of *FT* have also been extensively described in woody perennial trees [22–26]. However, genetic research and conventional breeding are limited by the long juvenile period of woody plants. To shorten the generation time, several fast-track breeding systems have been developed for woody plants. For example, a rapid breeding system was developed to transform the citrus tristeza virus resistance of trifoliate orange into citrus germplasm through the overexpression of Satsuma mandarin *FT* in trifoliate orange [22, 55]. A previous study also found that expression of *AtFT* or *CiFT* using a Citrus leaf blotch virus-based vector could successfully promote the phase transition in juvenile citrus plants [36]. A recent study reported that a chimeric FT protein with moderate expression could promote citrus flowering without negatively affecting the edible parts of citrus [37]. Similarly, overexpression of *AtFT* in eucalyptus resulted in very early flowering, and this is also known as an effective way to accelerate eucalyptus breeding and genetic improvement [56]. Therefore, early flowering conferred by rootstocks carrying florigen reduces the generation time of woody plants by grafting or crossing, and this could provide a helpful and safe tool to dramatically accelerate citrus genetic studies and breeding.

Overexpression of trifoliate orange and *Arabidopsis FT* in tomato led to an early flowering phenotype in this study, similar to the overexpression of tomato *SFT* [12]. Grafting experiments with non-transgenic tomato grafted onto *35S:ToFT* and *35S:AtFT* transgenic rootstocks resulted in early flowering of non-transgenic scions with no detectable *ToFT* and *AtFT* mRNA, and ToFT and AtFT proteins were detected in the scions, consistent with findings in annual plants [12, 16, 17, 54, 57]. These results suggest that FT proteins from woody plants can move in annual plants. Grafting experiments in trifoliate orange showed that ToFT and AtFT proteins can also move over long distances from rootstocks to scions. These data further indicate that FT transport is conserved between annual and perennial plants. However, *FT* mRNA can also be transmitted in *Arabidopsis* and tomato–tobacco heterografts [11, 43], implying that although the transport of FT protein is conserved, the transport of mRNA may be species-specific. In previous studies on the transport of FT in woody plants, all experiments focused on the transport of FT from the rootstock to the scion and ignored the possibility of FT transport from the scion to the rootstock. Here, we found that FT protein was transported both upwards and downwards in grafting experiments. These results imply that FT protein transport not only occurs over a long distance but also is bi-directional. Recently, FT with a light-induced TGACG-motif binding factor was reported to move from the shoot to the root under light, and together, they formed a signaling module in the root to induce the expression of soybean nodulation factors [58]. Furthermore, the *FT* homolog *StSP6A* controls flowering and storage organ formation in potato [59]. These results suggest that *FT* may also play an important role in the root development of trifoliate orange.

Wild-type tomato scions were grafted onto *35S:AtFT* and *35S:ToFT* transgenic tomato rootstocks to promote early flowering of the scions. However, when *35S:ToFT* and *35S:AtFT* transgenic trifoliate orange were used as rootstocks, the transport of FT protein did not significantly change the flowering time of non-transgenic scions during the 2 years of observation. Furthermore, our observations are consistent with the results of grafting experiments in other woody plants such as cassava, poplar, apple, and plum, in which transgenic rootstocks overexpressing *FT* did not promote the flowering of non-transgenic recipient scions [23, 28, 32, 60]. Recently, a report showed that a transgenic Citrange rootstock in which *CiFT3* is driven by a phloem-specific promoter (SUCROSE SYNTHASE 2, SUC2) can promote the flowering of grafted juvenile scions [40]. However, we used the same citrus variety for the scion and the transgenic rootstock in this study, and an early flowering phenotype was not observed in the grafted scion. We speculate that this phenomenon may be caused by different promoters; the constitutive 35S promoter was used in our study, whereas a phloem-specific SUC2 promoter was used in the previous study [40]. Previous studies have indicated that the *SUC2* promoter may be advantageous for floral induction because it drives transgene expression of graft-transmitted FT protein in woody plants [30, 40].

In Carrizo citrange and *Jatropha*, *FT* transgenic shoots with flower buds driven by the *35S* strong promoter did not develop into normal transgenic plants [30, 40], whereas healthy transgenic trifoliate orange shoots were obtained when we used the *35S* promoter and grafted onto rootstocks of WT seedlings in this study. Therefore, we speculate that in addition to different promoters, there may be other reasons for this phenomenon. First, there is a maturity gradient in the meristems of woody plants, and there seems to be a threshold number of FT products required for flower induction. The lack of early flowering in trifoliate orange grafts may be caused by dilution or attenuation of FT products during long-distance transport to the non-transgenic recipient scion through the graft junction. Second, the flowering of woody plants is an extraordinarily complicated process that requires the regulation of multiple genes [6, 35, 61–64]. Interlocking feedback loops may regulate the dynamic behavior of the floral transition in trifoliate orange [65, 66]. For example, FT protein transported to the scion may inhibit the expression of endogenous FT proteins. Third, coordinated transport of multiple FT and FT-like proteins may be required to promote flowering in grafted trifoliate orange. For example, there are three highly similar *FT* homologs (*CiFT1*, *CiFT2*, and *CiFT3*) in Satsuma mandarin. Further expression and transgenic analysis revealed that *CiFT2* and *CiFT3* are involved in floral induction [38] and flowering [22, 37, 40]. We searched the trifoliate orange genome database, and two other homologs (Pt6g020010 and Pt6g016100) were identified in addition to *ToFT*. Finally, the juvenile period of wild-type trifoliate orange is usually 6–8 years. However, tomato only takes 2–3 months from seed germination to flowering. Thus, the transported FT protein was able to promote the flowering of wild-type tomato scions in the grafting experiment, but it could not promote the flowering of wild-type citrus scions.

In model plants, the transport of FT from leaves to the SAM is mediated by some FT interaction partners [19–21]. FTIP1 plays an indispensable role in the FT protein transport of *Arabidopsis* and rice [19, 21]. In trifoliate orange, *ToFTIP1* exhibited characteristics similar to those of *Arabidopsis FTIP1*. In addition to sequence similarity between *ToFTIP1* and *AtFTIP1* or *OsFTIP1*, *ToFTIP1* is specifically expressed in vascular tissues and presents an expression pattern similar to that of *ToFT*, indicating that *ToFTIP1* acts similarly in trifoliate orange and model plants [19, 21, 67]. A recent report confirmed the interaction between NnFTIP1 and NnFT1 and showed that NnFTIP1 influenced NnFT1 transport from the leaves to the apical meristem of lotus [68]. Furthermore, both ToFT and ToFTIP1 were colocalized in the ER in tobacco cells, implying that ToFTIP1 may interact with ToFT. Subsequently, several approaches confirmed the interaction between ToFTIP1 and ToFT. The tomato transformation experiment further confirmed that *ToFTIP1* can participate in trifoliate orange flowering. These observations support the view that ToFTIP1 is the trifoliate orange counterpart of FTIP1 and interacts with ToFT to affect flowering time in trifoliate orange. Interestingly, overexpression of *ToFTIP1* induced the upregulation of *SFT* in transgenic tomato. These results are consistent with observations in *Arabidopsis*. A previous study showed that the expression of *FT* was downregulated in the *ftip1–1 Arabidopsis* mutant [19]. These results suggest that *ToFTIP1* may require the participation of *FT* to promote flowering in transgenic plants. However, further studies are required, as this is a preliminary study.

Based on the above research, AtFT and ToFT can promote the flowering of transgenic tomato and trifoliate orange. The flowering of transgenic plants may be caused by exogenous FT protein directly forming a complex with FD to promote the expression of downstream genes [53, 69]. ToFT and AtFT can be transported in tomato and trifoliate orange. However, *FT*-transgenic trifoliate orange rootstocks cannot promote the flowering of grafted juvenile scions because the transported ToFT protein may inhibit endogenous ToFT protein and cause low accumulation in trifoliate orange. In this study, ToFTIP1 interacted with ToFT, similar to results in *Arabidopsis* and rice [19, 21]. Thus, we speculated that ToFTIP1 may play a role specifically in FT transport from companion cells to sieve elements, based on research in model plants [19, 21]; this in turn promotes the expression of *AP1* and finally leads to trifoliate orange flowering. However, further research is needed to understand the mechanism by which exogenous FT protein leads to a reduction in endogenous FT protein in trifoliate orange. Furthermore, it remains to be determined whether this phenomenon is specific to the juvenile period of trifoliate orange and whether seasonal flowering can be overcome if adult trifoliate orange scions are grafted onto *FT* transgenic rootstocks.

## Experimental procedures

### Plant material and growth conditions

Transgenic trifoliate orange (*C. trifoliata*) and tomato were grown in a glasshouse at 60%–75% relative humidity and ambient temperature (>20°C) under natural daylight at the National Citrus Breeding Center of Huazhong Agricultural University, Wuhan, China. *AtFT* and *ToFT* sequences were amplified from mature leaves of trifoliate orange and *Arabidopsis* with Phanta Max Super-Fidelity DNA Polymerase (Vazyme, P505-d1). Their coding sequences (CDSs) were fused with the GFP protein under the control of the 35S promoter in the pRIE101 vector using the ClonExpress II One Step Cloning Kit (Vazyme, C112–01). The primers used are shown in Table S1. The fusion constructs (*35S:ToFT-GFP* and *35S:AtFT-GFP*) and control vector (*35S:GFP*) were introduced into *Agrobacterium tumefaciens* GV3101 using the freeze–thaw method. All constructed vectors were confirmed by sequencing. Transgenic trifoliate orange with *ToFT*, *AtFT*, and *GFP* (pRIE101 empty vector) were obtained by *A. tumefaciens*-mediated transformation of epicotyls based on a previously reported method [70]. Transgenic tomato with *ToFT*, *AtFT*, and *GFP* was generated from cotyledon explants based on a previously described method [71].

### Generating grafts

*ToFT*, *AtFT*, and *GFP* transgenic and wild-type plants were used for grafting experiments. Transgrafting included the following combinations: self-grafted wild-type plants, transgenic scions grafted onto wild-type rootstocks, and wild-type scions grafted onto transgenic rootstocks. For statistical analysis, at least 12 individual plants were required to survive for each grafting combination. The wild-type and transgenic trifoliate orange were grown in 50-cm plastic pots that contained a mixture of perlite and commercial medium (1:3), and the plant heights ranged from 1 to 1.5 m. Grafts of trifoliate orange were obtained by bud grafting onto rootstocks. At least one branch below the graft union on the rootstock was maintained for each graft.

One-month-old wild-type and transgenic tomato plants were grafted by the wedge-shaped grafting method. The scions were cut into a wedge from 8 cm below the apex and then inserted into the rootstock with a vertical cut after removing the apex. For each graft, at least two leaves below the graft union on the rootstock were maintained. The graft unions were fixed with Parafilm (Pechiney Plastic Packaging Company), and the grafted plants were placed in sealed bags to maintain moisture for one week. The bags were ventilated daily and removed when the graft leaves had enlarged. Mature scion leaves on successfully grafted plants were periodically removed to ensure sink strength. The date of flower initiation was recorded when the first floral buds were visible. GFP fluorescence in plant tissues was observed using the NightSHADE LB 985 system (Berthold, Bad Wildbad, Germany).

### Western blot analysis

To determine the transport of ToFT/AtFT-GFP protein, the SAM and leaves of new shoots from the scion and rootstock of grafts were extracted from below and above the graft unions. Total protein was isolated using extraction buffer consisting of 25 mM Tris–HCl (pH 7.8), 2.5 mM MgCl_2_, 100 mM KCl, 0.1% Nonidet P40, 0.2% Tween-20, 1 mM DTT, and cOmplete Protease Inhibitor Cocktail (Roche) as described previously [72]. Equal amounts of total protein were separated by SDS–PAGE, and then ToFT/AtFT-GFP protein was incubated with anti-GFP antibody (3F10, Roche) and analyzed by western blot detection. Accumulation of the transported FT protein was relatively low in the trifoliate orange scions, and the total extracted protein was therefore enriched using an Anti-GFP Magarose Beads Kit (Smart-Lifesciences, China) and used for western blotting.

ToFT protein was detected using an anti-ToFT antibody (GenScript, Nanjing, China). The ToFT antibody was prepared and tested as previously described [73]. The CDS of *ToFT* was cloned into the pET-28a vector. ToFT protein was purified from *Escherichia coli* strain BL21 (DE3) using a Ni^2+^ column. For primary immunization, 1 mg of the ToFT-HIS protein and the same volume of Freund’s complete adjuvant (Sigma-Aldrich) were mixed and emulsified at multiple points on the back of two healthy New Zealand rabbits. Then, reimmunization was conducted at biweekly intervals with 0.5 mg of the ToFT-HIS protein mixed with incomplete Freund’s adjuvant. Blood was taken one week after the third immunization, and then the antibody protein was precipitated with 50% saturated ammonium sulfate. Later, the pellet was removed by dialysis and dissolved in 10 mM PBS (pH 7.4). The obtained IgG antibodies were purified on a column with protein A-agarose beads equilibrated with the same PBS buffer and were eluted with 100 mM glycine buffer (pH 3.0). The fractions containing IgG were combined, and the pH was adjusted to 7.4. Antiserum titer was determined by ELISA.

### Expression analysis

Total RNA from the SAM and leaves was extracted using the TRIzol reagent (Invitrogen, Carlsbad, CA, USA). The PrimeScript RT Reagent Kit with gDNA Eraser (TaKaRa, Dalian, China) was used for reverse transcription according to the user manual. qRT-PCR was performed according to the instructions of the qPCR SYBR Green Master Mix (Yeasen, Shanghai, China). The relative gene expression levels were normalized to the expression of *actin* based on a previously reported method [51]. The primers used for qRT-PCR are shown in Table 1. The PCR amplification procedure was as follows: 95°C for 5 min; 40 cycles of 95°C for 10 s, 59°C for 20 s, and 72°C for 20 s.

### Yeast two-hybrid assay (Y2H)

To identify proteins that interacted with ToFT during long-distance movement, a Y2H assay was performed using the Matchmaker Gold Yeast Two-Hybrid System (TaKaRa, Dalian, China). Stems of trifoliate orange were collected and used to construct a Y2H library. The ToFT coding sequence (CDS) was inserted into the pGBKT7 vector and transformed into the Y2H Gold yeast strain Y187. The transformed cells were selected on SD–histidine (His)/−tryptophan (Trp)/−leucine (Leu)/−adenine (Ade) medium supplemented with 40 μg/ml X-a-Gal. To further verify the interaction of FT (ToFT and AtFT) with its potential interacting proteins (ToFTIP1 and SlFTIP1), their CDSs were cloned into the pGADT7 vector and then transformed into yeast AH109.

### Subcellular localization and bimolecular fluorescence complementation assay (BiFC)

To determine the subcellular localization of ToFTIP1 and ToFT, the IntF2A-based polyprotein transgene system was used [50], and the CDSs of *ToFTIP1* and *ToFT* without termination codons were fused to the 5′ ends of genes encoding red fluorescent protein (RFP) and GFP, respectively. The p35S-SPYNE and p35S-SPYCE vectors with the N terminus of YFP and the C terminus of YFP, respectively, were used to construct the target proteins and their interaction partners for BiFC assays. These fusion constructs and the control were integrated into *Agrobacterium* GV3101 and then co-infiltrated into tobacco leaves, which were observed using a confocal microscope (TCP SP5, Leica Microsystems) as described previously [74].

### Glutathione S-transferase (GST) pull-down assay

To obtain GST and His-tagged proteins, the CDSs of target genes were inserted into the pGEX-6p-2 vector (Pharmacia, Uppsala, Sweden) and the pET32a vector (Novagen, Madison, WI, USA). These constructs and the control vector were transformed into *E. coli* strain BL21 (DE3). Then 0.2 mM isopropyl-β-d-thiogalactopyranoside (IPTG) was added to the medium to induce the formation of fusion proteins, and the cells were cultured at 28°C for 4 hours. The supernatants containing the GST recombinant protein and His recombinant protein lysates were blended in glutathione sepharose beads (TransGen Biotech, Beijing, China). The eluted proteins were detected by western blot analysis with anti-GST and anti-His antibodies for GST, GST-ToFT/AtFT, His, and His-ToFTIP1/SlFTIP1 proteins. The experimental methods were described previously [75].

### Statistical analysis

Significant differences (P < 0.05) were analyzed using one-way analysis of variance (ANOVA) followed by t-tests in MS Excel 2016 (Microsoft). ^*^P < 0.05, ^**^P < 0.01.

### Ethics declarations

None.

## Data availability

The data that support the findings of this study are available from the corresponding author upon reasonable request.

## Conflict of interest

The authors declare that they have no conflict of interest.

## Supplementary data

Supplementary data is available at *Horticulture Research* online.

## Supplementary Material

Web_Material_uhac056
