# Supplementary material for: Mobility of FLOWERING LOCUS T protein as a systemic signal in trifoliate orange and its low accumulation in grafted juvenile scions
Source: Hortic Res. 2022 Mar 7;9:uhac056. doi: 10.1093/hr/uhac056 (PMC9186307; doi:10.1093/hr/uhac056)
Supplement: Web_Material_uhac056 [file web_material_uhac056.zip › Figure S1-10(2).pdf]

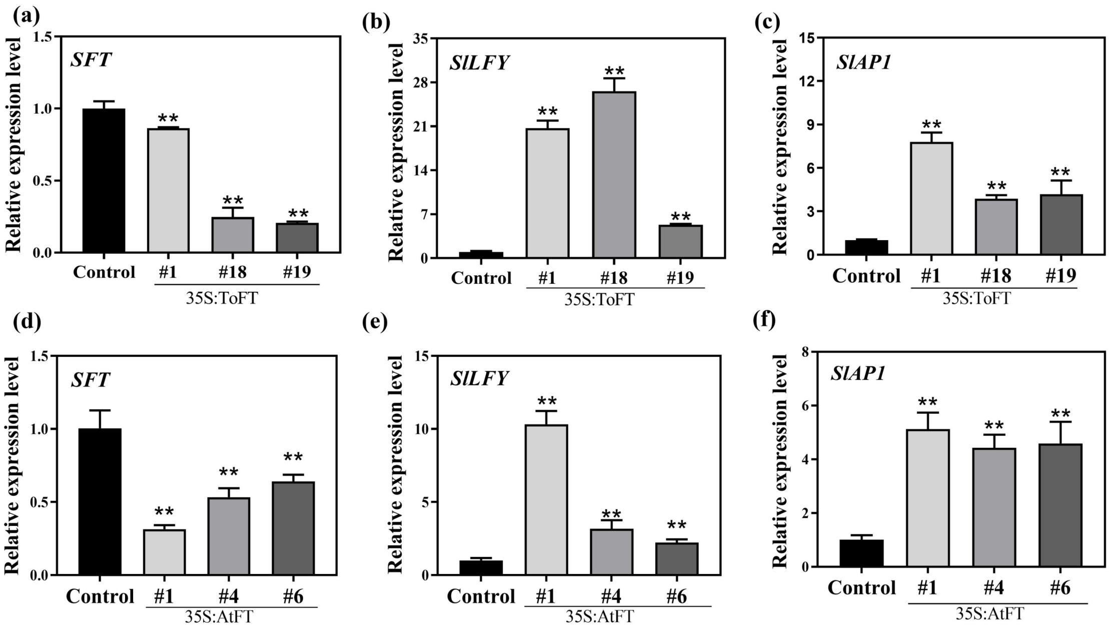

Figure S1 Expression analysis of *SFT*, *SlLFY*, and *SlAP1* in transgenic tomato. (a-c) The expression analysis of *SFT* (a), *SlLFY* (b), and *SlAP1* (c) in *35S:ToFT* transgenic tomato and control plants. (d-f) The expression analysis of *SFT* (d), *SlLFY* (e), and *SlAP1* (f) in *35S:AtFT* transgenic tomato and control plants. Error bars represent ± SE (n=3) in all the panels.

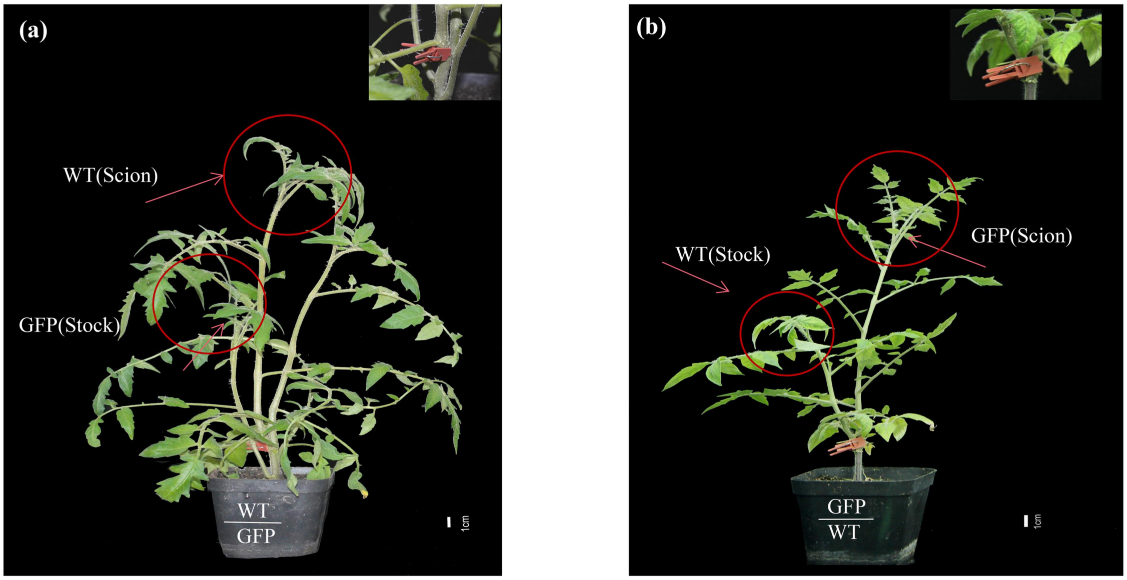

Figure S2 Demonstration of the graft and location of the collected sample in tomato. (a) A recipient wild-type scion was grafted onto a donor transgenic rootstock (*35S:GFP*). (b) A donor transgenic scion (*35S:GFP*) was grafted onto a recipient wild-type rootstock. The red plastic clip represents the joints of scions and rootstocks. The red circle represents the location of the sample collection. Bar = 1cm.

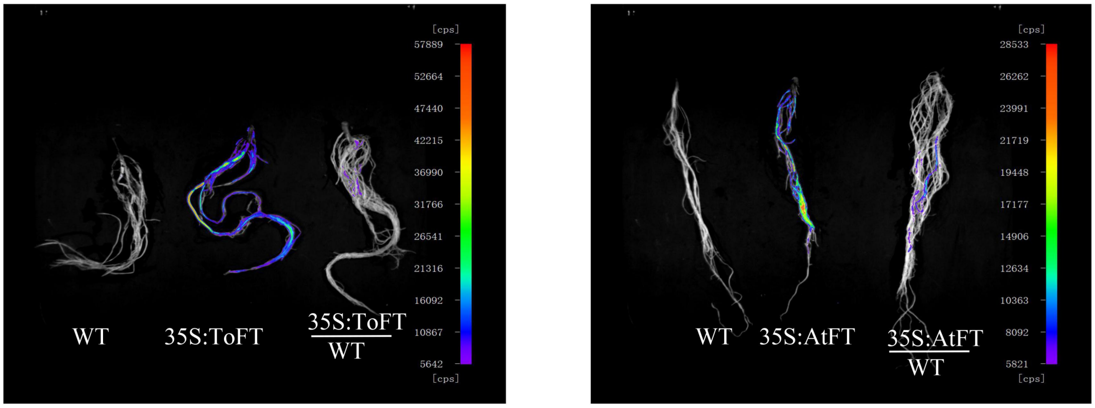

Figure S3 The NightSHADE LB985 imaging system detected the transfer of AtFT and CiFT proteins from transgenic scions to the roots of wild-type rootstocks in tomato.

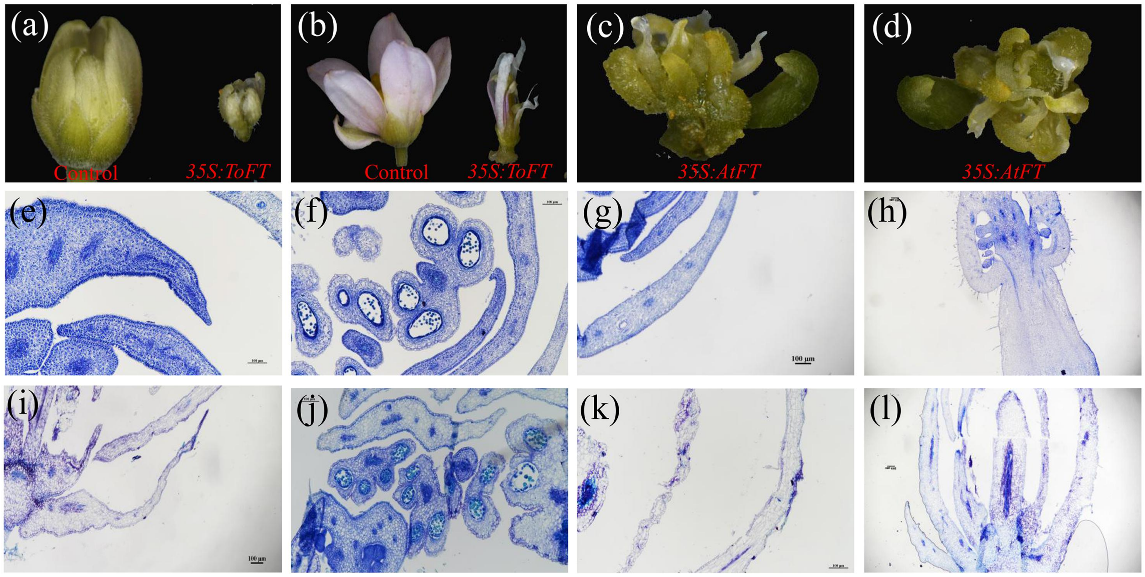

Figure S4 Morphological and cytological analysis of flowers of wild-type and extremely early-flowering transgenic trifoliate orange. (a-d) Morphological analysis of wild-type flowers and extremely early-flowering transgenic trifoliate orange. (e-g) Cytological observation of wild-type flowers. (e) Sepals, (f) anthers, (g) sepals, and (h) stigmas. (i-l) Cytological observation of flowers of extremely early-flowering transgenic trifoliate orange. (i) Sepals, (j) anthers, (k) petals, and (l) stigmas. Bar = 100 μm.

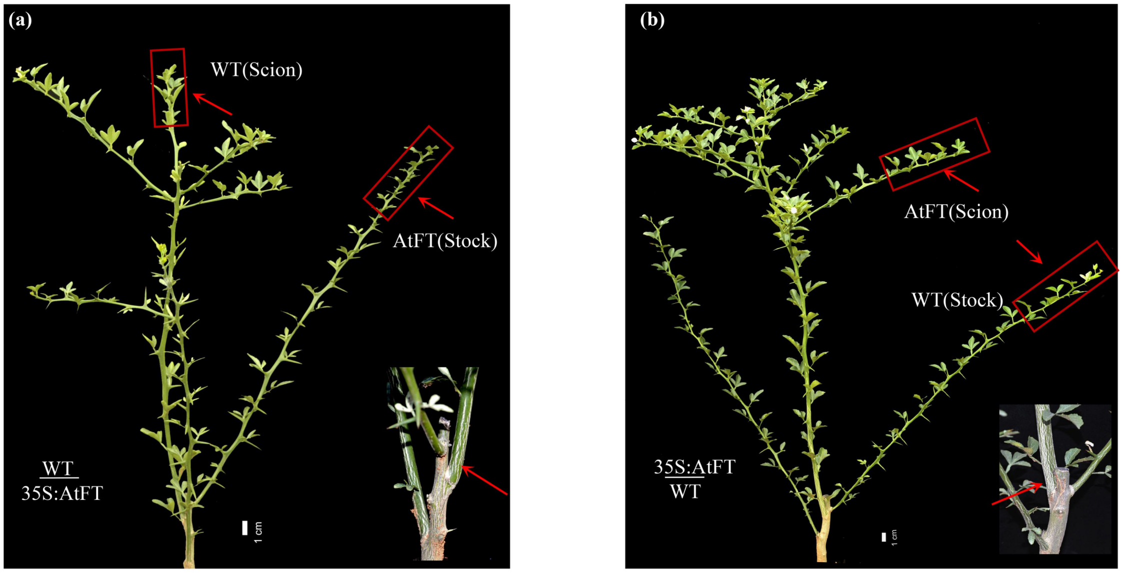

Figure S5 Demonstration of the graft and location of the collected sample. (a) A recipient wild-type scion was grafted onto a donor transgenic rootstock (*35S:AtFT*). (b) Demonstration of the graft and location of the collected sample. A donor transgenic scion (*35S:AtFT*) was grafted onto a recipient wild-type rootstock. The red circle represents the location of the sample collection. Bar = 1cm.

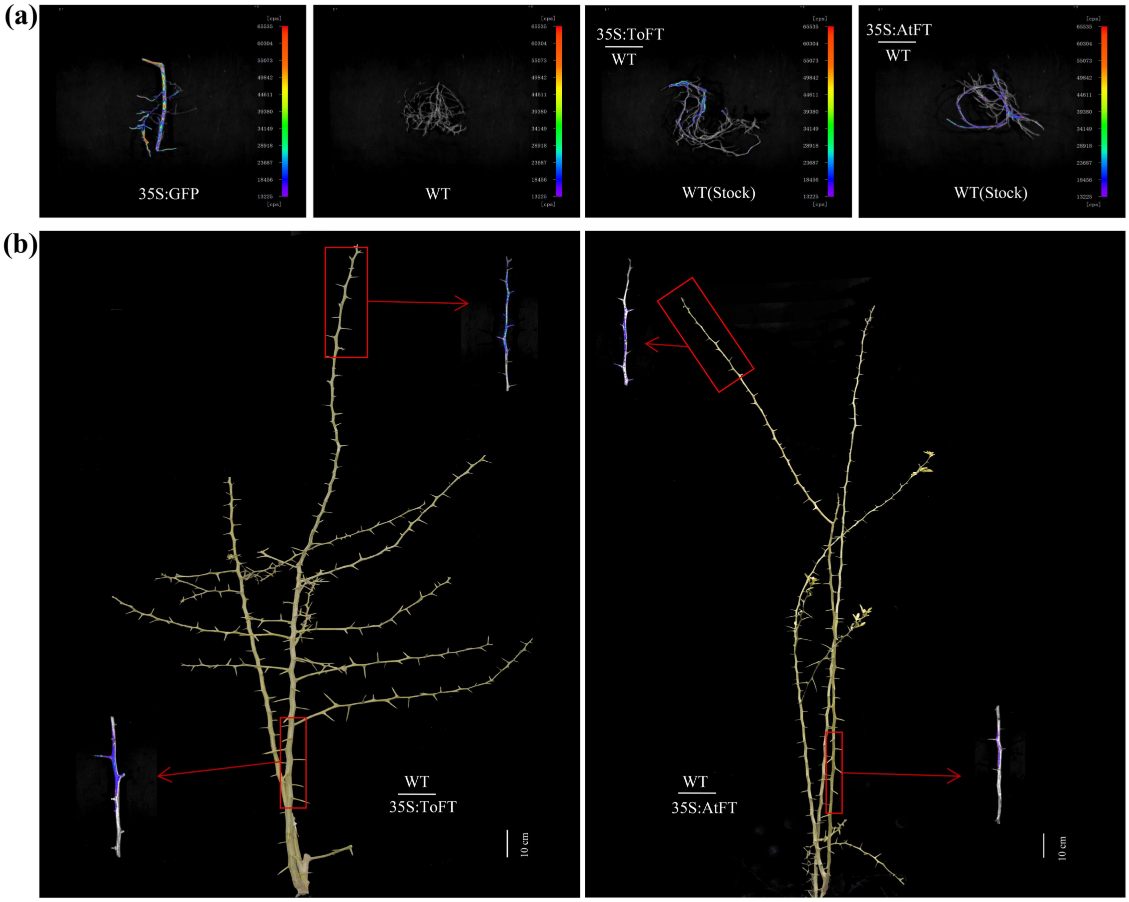

Figure S6 Graft transmissible ToFT and AtFT proteins underwent long-distance movement for systemic signaling. (a) The NightSHADE LB985 imaging system detected the transfer of AtFT and ToFT proteins from transgenic scions to the roots of wild type rootstocks in trifoliate orange. (b) Long-distance movement analysis of ToFT and AtFT proteins from transgenic trifoliate orange rootstocks to wild-type scions in winter. Bar = 10 cm.

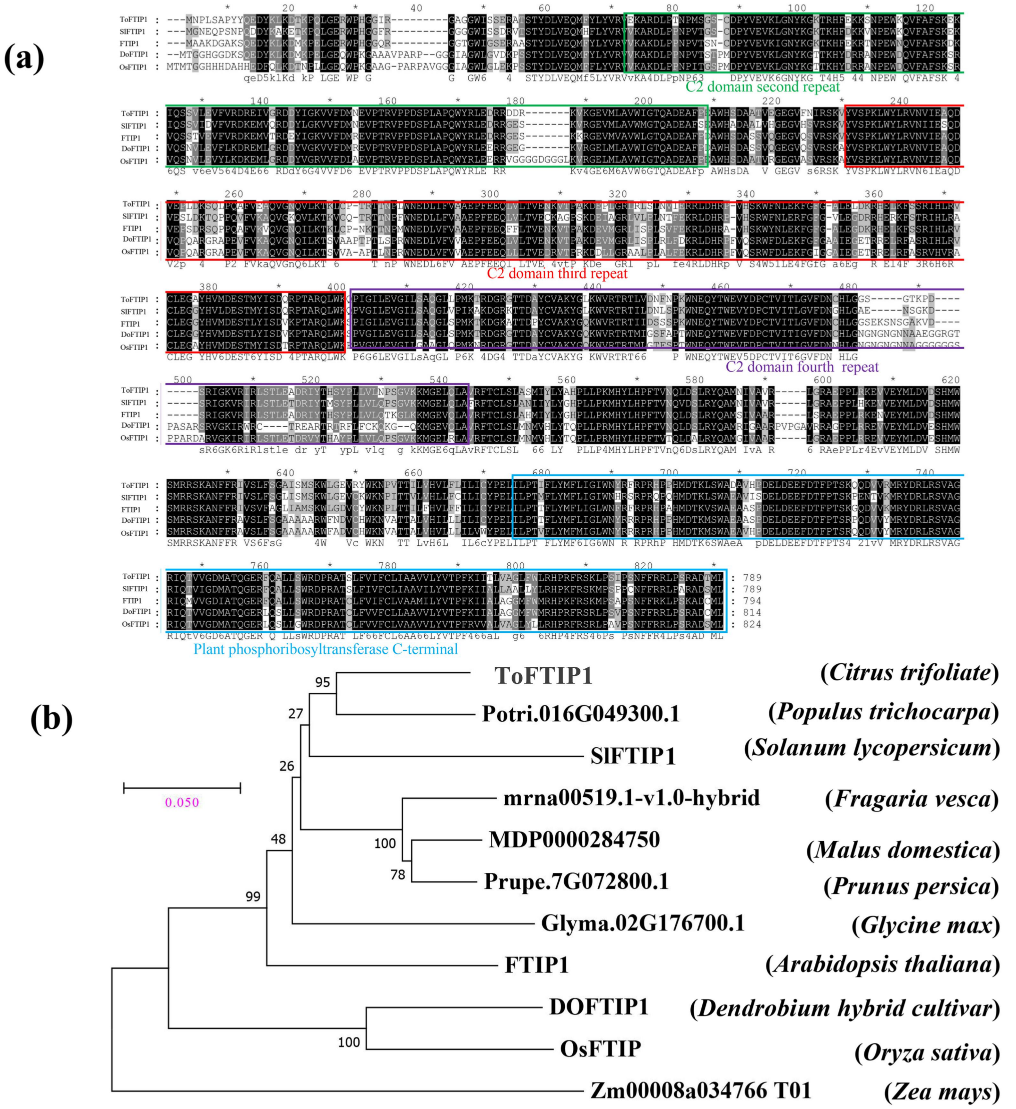

Figure S7 Gene structure, sequence alignments and phylogenetic analysis of *ToFTIP1*. (a) Multiple alignments of the ToFTIP1 and FTIP1 from other plants. (b) Phylogenetic analysis of ToFTIP1. MEGA7 software was used with the neighbor-joining (NJ) method, the Jones–Taylors–Thornton (JTT) model, and 1000 bootstrap replicates.

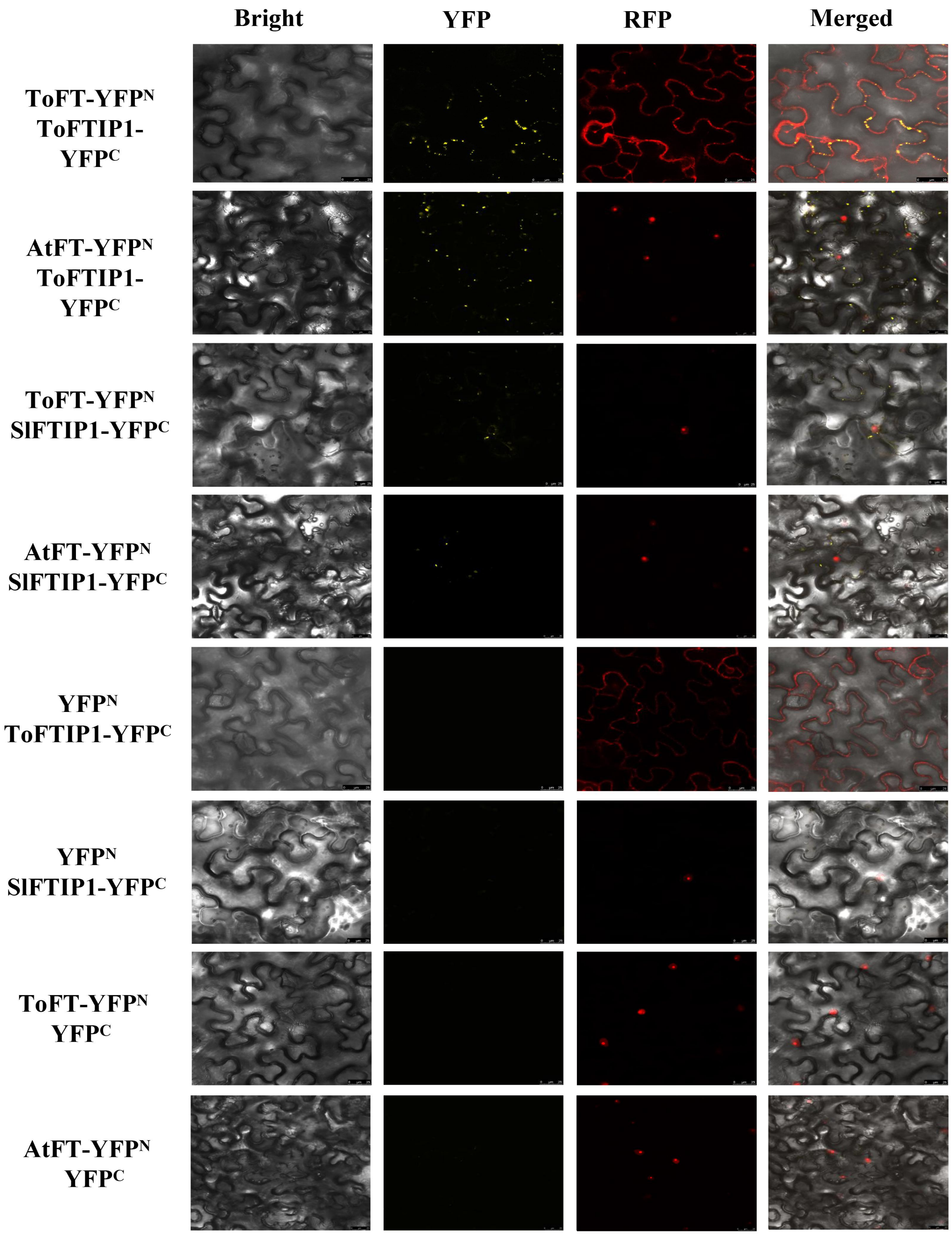

Figure S8 BiFC analysis of the interactions of ToFTIP1 and AtFT, ToFTIP1 and ToFT, SlFTIP1 and AtFT, and SlFTIP1 and ToFT in tobacco leaf cells. Red color indicates the fluorescence of nuclear marker (VirD2NLS-mCherry). Scale bars = 25 μm.

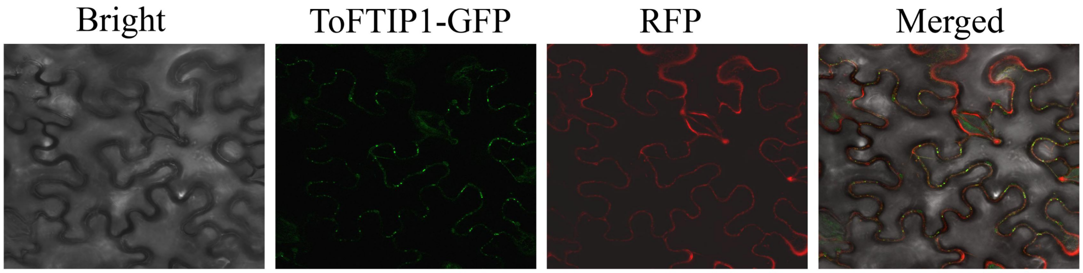

Figure S9 Subcellular localization of ToFTIP1:GFP and an endoplasmic reticulum marker in tobacco leaf cells. RFP, the marker with red fluorescence is localized in the nucleus. Bar = 25μm.

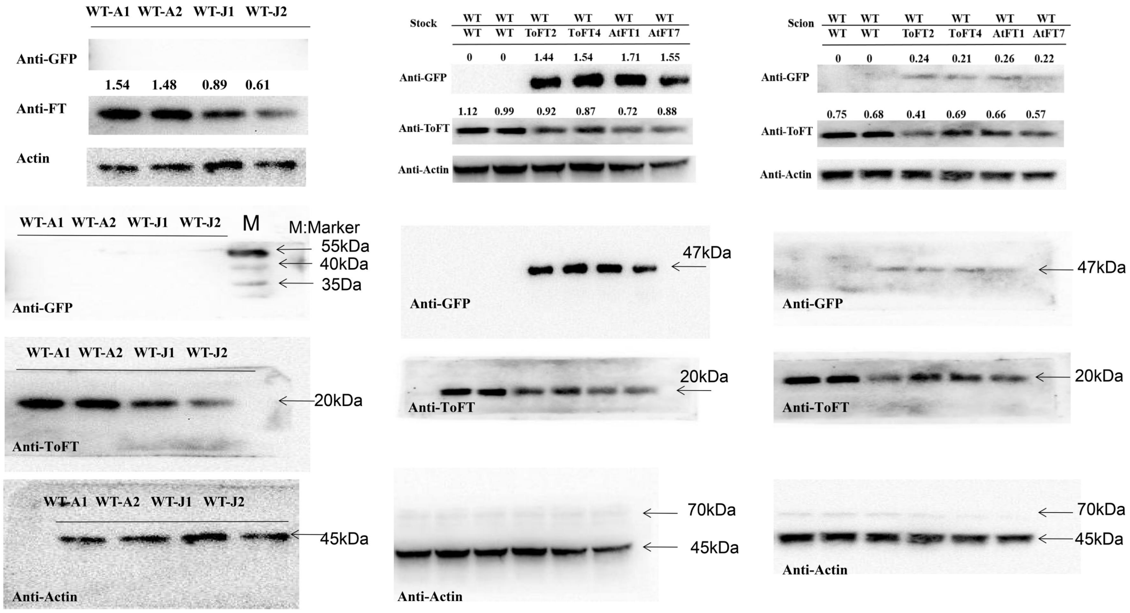

Figure S10 Original image of western blot in Figure 8.
